# *KLRC1* knockout overcomes HLA-E-mediated inhibition and improves NK cell antitumor activity against solid tumors

**DOI:** 10.3389/fimmu.2023.1231916

**Published:** 2023-08-21

**Authors:** Alice Mac Donald, Delphine Guipouy, William Lemieux, Mario Harvey, Louis-Jean Bordeleau, David Guay, Hugo Roméro, Yuanyi Li, Renaud Dion, Kathie Béland, Elie Haddad

**Affiliations:** ^1^ Centre Hospitalier Universitaire (CHU) Sainte-Justine Research Center, Montréal, QC, Canada; ^2^ Department of Microbiology, Infectiology and Immunology, University of Montréal, Montréal, QC, Canada; ^3^ Feldan Therapeutics, Québec, QC, Canada; ^4^ Department of Pediatrics, University of Montreal, Montreal, QC, Canada

**Keywords:** natural killer cells, inhibitory receptor, HLA-E, NKG2A, solid tumor, cytotoxicity

## Abstract

**Introduction:**

Natural Killer (NK) cells hold the potential to shift cell therapy from a complex autologous option to a universal off-the-shelf one. Although NK cells have demonstrated efficacy and safety in the treatment of leukemia, the limited efficacy of NK cell-based immunotherapies against solid tumors still represents a major hurdle. In the immunosuppressive tumor microenvironment (TME), inhibitory interactions between cancer and immune cells impair antitumoral immunity. *KLRC1* gene encodes the NK cell inhibitory receptor NKG2A, which is a potent NK cell immune checkpoint. NKG2A specifically binds HLA-E, a non-classical HLA class I molecule frequently overexpressed in tumors, leading to the transmission of inhibitory signals that strongly impair NK cell function.

**Methods:**

To restore NK cell cytotoxicity against HLA-E^+^ tumors, we have targeted the NKG2A/HLA-E immune checkpoint by using a CRISPR-mediated *KLRC1* gene editing.

**Results:**

*KLRC1* knockout resulted in a reduction of 81% of NKG2A^+^ cell frequency in *ex vivo* expanded human NK cells post-cell sorting. *In vitro*, the overexpression of HLA-E by tumor cells significantly inhibited wild-type (WT) NK cell cytotoxicity with *p*-values ranging from 0.0071 to 0.0473 depending on tumor cell lines. In contrast, *KLRC1* ^KO^ NK cells exhibited significantly higher cytotoxicity when compared to WT NK cells against four different HLA-E^+^ solid tumor cell lines, with *p*-values ranging from<0.0001 to 0.0154. Interestingly, a proportion of 43.5% to 60.2% of NKG2A^−^ NK cells within the edited NK cell population was sufficient to reverse at its maximum the HLA-E-mediated inhibition of NK cell cytotoxicity. The expression of the activating receptor NKG2C was increased in *KLRC1* ^KO^ NK cells and contributed to the improved NK cell cytotoxicity against HLA-E^+^ tumors. *In vivo*, the adoptive transfer of human *KLRC1* ^KO^ NK cells significantly delayed tumor progression and increased survival in a xenogeneic mouse model of HLA-E^+^ metastatic breast cancer, as compared to WT NK cells (*p* = 0.0015).

**Conclusions:**

Our results demonstrate that *KLRC1* knockout is an effective strategy to improve NK cell antitumor activity against HLA-E^+^ tumors and could be applied in the development of NK cell therapy for solid tumors.

## 1 Introduction

Due to their unique ability to recognize and kill tumor cells, Natural Killer (NK) cells have emerged as a promising option for adoptive cell transfer in cancer immunotherapy. Unlike T cell-based approaches, NK cells can be used safely in an allogenic setting, therefore holding high potential for off-the-shelf cellular therapy. Recently, the antitumor activity of CAR-NK cells has been harnessed in the clinic in an allogenic setting with encouraging outcomes for the treatment of hematologic malignancies (1). However, the efficacy of both T and NK cells in treating solid tumors has been limited (2). One reason for this limitation is the impaired cytotoxic function of NK cells due to the immunosuppressive tumor microenvironment (TME).

NK cell cytotoxicity depends on the integration of both activation and inhibition signals through the engagement of a variety of receptors, providing a range of powerful means to eliminate malignant cells. Upon NK cell contact with a tumor cell, the lack of interactions between MHC molecules and inhibitory receptors, combined with signals from activating receptors, synergistically shifts the balance toward NK cell activation, resulting in target cell lysis. Although the dominance of inhibitory over activating signals is crucial for NK cell self-tolerance, this negative regulation can be exploited by tumor cells to escape the NK cell response.

The NKG2A/HLA-E axis is a prominent inhibitory checkpoint that impairs NK cell antitumor activity, particularly in solid malignancies. IFN-γ, secreted in the TME, promotes HLA-E expression on the tumor cell surface, which binds its specific receptor NKG2A, a major NK inhibitory receptor, encoded by the *KLRC1* gene (3, 4). HLA-E is a non-classical MHC class I molecule that displays peptides derived from the signal peptides of other classical MHC-I molecules. While HLA-E is ubiquitously expressed at low levels at the cell surface, its overexpression has been reported in various solid cancer types. An analysis of 10,375 human tumors, representing 33 tumor types, revealed that HLA-E is widely overexpressed by tumor cells (5). Moreover, tumor-infiltrating NK cells are predominantly NKG2A^+^, and the high density of those cells has been correlated with worse survival in patients with solid cancer (6–8).

To develop NK cells that are resistant to HLA-E-mediated inhibition, we generated *KLRC1-*knockout human NK cells (*KLRC1* ^KO^ NK) by using the CRISPR technology. The present study compares the antitumor activity of *KLRC1* ^KO^ and wild-type (WT) NK cells against several HLA-E^+^ solid tumor cell lines *in vitro* and in an HLA-E^+^ breast cancer xenograft mouse model.

## 2 Methods

### 2.1 Cell culture and cell lines

K562-mb-IL21 feeder cells (9), kindly given by Dean A. Lee (Nationwide Children’s Hospital), were cultured in complete Roswell Park Memorial Institute (RPMI) media, composed of RPMI supplemented with 10% fetal bovine serum (FBS), penicillin (100 units/mL), and streptomycin (100 µg/mL), and maintained between 0.1 × 10^6^ and 1 × 10^6^ cells/mL. Human breast adenocarcinoma cell line MDA-MB-231 (ATCC, Manassas, VA, USA; HTB-26™), lung carcinoma A549 (ATCC, CCL-185™), and colorectal adenocarcinoma HT-29 (ATCC, HTB-38™) were cultured in complete Dulbecco’s modified Eagle medium (DMEM) media. Breast adenocarcinoma cell line SK-BR3 (ATCC, HTB-30™) was cultured in complete RPMI media. 293T cells (ATCC, CRL-3216™) were cultured following ATCC recommendations. Culture media were purchased from Gibco (Grand Island, NY, USA): RPMI 1640 (11875093) and DMEM (11995065). FBS (080150) and Penicillin-Streptomycin Solution (450-201-EL) were purchased from Wisent (St-Bruno, QC, Canada).

### 2.2 Molecular cloning

The pHUS-Luciferase-eGFP vector was constructed in our laboratory by adding a UCOE sequence (10) to the pHRSIN-SFFV-eGFP (11) upstream of the SFFV promoter to produce pHRSIN-UCOE-SFFV-eGFP. In addition, firefly luciferase and internal ribosome entry site (IRES) sequences were added upstream of the eGFP sequence, resulting in a pHRSIN-UCOE-Luc-IRES-eGFP vector. The pHUS-HLA-E-Cw1502 PGK BSD vector was generated by cloning the cDNA of the HLA-E*010301 allele, which was obtained via gene synthesis from Integrated DNA Technologies (IDT; Coralville, IA, USA), first into pENTR1a gateway entry 1A vector (Addgene, Cambridge, MA, USA; 17398). Then, the HLA-E signal peptide was replaced by the nonameric peptide derived from the HLA-Cw1502 signal sequence (VMAPRTLLL). The entire sequence was cloned by gateway LR reaction in the pHRSIN-UCOE.0.7-SFFV-DEST-PGK-BSD encoding for the UCOE, the SFFV promoter, and the blasticidin resistance gene under the PGK promoter that we modified from the lentiviral vector pHRSIN-SFFV, which was a gift from Els Verhoeyen Lab.

### 2.3 Lentiviral transduction of tumor cell lines

All the tumor cell lines were modified to express luciferase and green fluorescent protein (GFP) by lentiviral transduction with pHUS-Luciferase-eGFP. HLA-E overexpression was induced by lentiviral transduction in Luciferase-GFP^+^ tumor cell lines using the pHUS-HLA-E-Cw1502 PGK BSD vector. VSV-G pseudotyping lentiviral particles were generated by transfection of 293T cells as previously described (12). Viral titration was performed on 293T cells. Tumor cell line transductions were performed at a multiplicity of infection (MOI) of 1 by co-incubating cells with lentiviral particles for 24 hours. Lentiviral transduction of pHUS-HLA-E-Cw1502 PGK BSD was followed by a blasticidin (Invitrogen, Carlsbad, CA, USA; R21001) selection at 5 μg/mL. Clonal cell lines were obtained by a single cell sorting using flow cytometry with an AriaII cell sorter (BD Biosciences, San Jose, CA, USA), based on GFP and/or HLA-E expression.

### 2.4 NK cell isolation and culture

Blood samples were obtained after healthy volunteers provided informed consent (institutional review board (IRB)-approved protocol #CER-2019-1956). Peripheral blood mononuclear cells (PBMCs) were isolated by centrifugation on a Ficoll Paque Plus (Cytivia, Marlborough, MA, USA; 17144003) layer and washed in Dulbecco’s phosphate-buffered saline (DPBS) (Gibco, 14190144). Primary NK cells were expanded using the NK Amplification and Expansion System (NKAES) with irradiated K562-mb-IL21 feeder cells (100 Gy) as previously described (9). Briefly, PBMCs were co-cultured with irradiated K562-mb-IL21 feeder cells in complete RPMI supplemented with 40 IU/mL of IL-2 (Proleukin, Novartis Pharmaceuticals, Montreal, QC, Canada) for 1 week. After the first week of expansion, the remaining CD3^+^ cells were removed with the EasySep™ Human CD3 Positive Selection Kit II (STEMCELL Technologies, Vancouver, BC, Canada; 17851). Cell purity expansion was assessed by flow cytometry. The protocol for re-expanding NK cells for additional weeks remained identical, except for an increase in IL-2 concentration from 40 IU/mL to 100 IU/mL.

### 2.5 Generation of *KLRC1* ^KO^ NK cells

*KLRC1* ^KO^ NK cells were generated by using the CRISPR technology in NKAES cells. NK cells were isolated from the PBMCs of six different healthy donors and expanded for 1 week before CD3^+^ cell depletion. *KLRC1* knockout using Feldan Shuttle delivery was performed the day after CD3 depletion. A crRNA (gRNA) was designed to target the following sequence of *KLRC1* gene exon 2: 5′-ACTCAGACCTGAATCTGCCCC-3′. The gRNA with 2′-*O*-methyl modifications at both ends was purchased as “100 nM RNA Oligo” from IDT ([mC]*U*UAAUUUCUACUCUUGUAGAUGGGGCAGAUUCAGGUCUGA*G*[mU]). The * indicates phosphorothioate bonds. The nuclease MAD7 (3.2 µM) and gRNA (4 µM) were co-incubated in sterile DPBS for 5 minutes at room temperature. The complexed ribonucleoprotein (RNP) was delivered using the Feldan Shuttle peptide (10 µM) in an equal ratio, in expanded NKAES cells as previously described (13). Briefly, NKAES cells were washed in DPBS and resuspended in 100 µL of RNP : Shuttle peptide mix. The mix was incubated for 90 seconds at room temperature before the addition of 400 µL of RPMI complete media. After centrifugation, cells were resuspended in RPMI complete medium with 100 IU/mL of IL-2. After Feldan Shuttle delivery, *KLRC1* ^KO^ NK cells were expanded with irradiated K562-mb-IL21 feeder cells for a culture period ranging from 2 to 6 weeks before fluorescence-activated cell sorting (FACS) based on loss of NKG2A expression. Subsequently, sorted NK cells were further expanded for at least an additional week and up to 6 weeks before being used in experiments. When utilizing frozen stocks, the thawed cells were expanded for a minimum of 1 week before their utilization. The nuclease MAD7 and the Shuttle peptide were provided by Feldan Therapeutics (Québec, QC, Canada). As an alternative to RNP delivery, a CRISPR/Cas9-expressing lentiviral vector was used to knock out *KLRC1* gene in NKAES cells from two other healthy donors. The gRNA sequences targeting *KLRC1* exon 2 (5′-AGGAGTAATCTACTCAGACC-3′ and 5′-AGGCAGCAACGAAAACCTAA-3′) and exon 3 (5′-GAAGCTCATTGTTGGGATCC-3′) were cloned into the Cas9 encoding plasmid plentiCRISPRv2 (Addgene, 52961), prior to production of Baboon envelope pseudotyped lentivirus. NK cells were transduced as previously described, and transduced cells were enriched in culture by puromycin selection (12). To assess the efficacy of gene edition, genomic DNA was extracted using DNeasy Blood & Tissue Kit (Qiagen, Hilden, Germany; 69504), and the targeted region was amplified by PCR using the primer sets 5′-TCACCCTTTTAATTGCACTAGGG-3′, 5′-AGCTTCTCTGGAGCTGATGG-3′ and purified using Monarch® PCR & DNA Cleanup Kit (NEB, Ipswich, MA, USA; T1030S). Genome editing efficiency was assessed using the T7 endonuclease I assay (T7E1; NEB, M0302S), and band densities were evaluated with FIJI distribution of ImageJ, using “Analyze gels” built-in functions.

### 2.6 Flow cytometry

All cell surface stainings were performed in ice-cold DPBS 2% FBS, 0.1% NaN_3_ sodium azide (Sigma, St. Louis, MO, USA; S2002) with a 30-minute incubation of antibodies at 4°C. Tumor cell lines were stained with the following antibodies: HLA-E-PE.Cy7 (BioLegend, 342607) or mouse IgG1 κ isotype control antibody (BioLegend, 400125). NK cells were stained with the following antibodies after isolation from PMBCs, for expansion and cell sorting: CD56-APC (BioLegend, 362503), CD3-FITC (BioLegend, 300440), and NKG2A-PE (Miltenyi, Bergisch Gladbach, Germany; 130-114-092). Dead cells were excluded by gating on 7-AAD^−^ (BD Biosciences, 559925). NK cells were stained with the following antibodies for phenotyping: CD56-FITC (BioLegend, 304603), NKG2A-PE (Miltenyi, 130-114-092), NKG2C-APC (Miltenyi, 130-130-663), KIR2D-APC (Miltenyi, 130-099-649), NKp44-APC (BioLegend, 325109), TGIT-APC (BioLegend, 372705), CD62L-APC (BioLegend, 304809), CD94-BV421 (BD Biosciences, 743948), FasL-BV421 (BioLegend, 306411), PD-1-BV421 (BioLegend, 329919), CXCR2-BV421 (BD Biosciences, 744195), NKp46-BV421 (BioLegend, 331913), NKp30-BV785 (BioLegend, 325229), CD57-BV785 (BioLegend, 393329), CX3CR1-BV785 (BioLegend, 341627), LAG-3-BV785 (BioLegend, 369321), NKG2D-BV785 (BioLegend, 320829), CD3-BUV395 (BD Biosciences, 564000), and CD16-BUV737 (BD Biosciences, 612786). Dead cells were excluded by gating on negative cells for LIVE/DEAD™ Fixable Blue (Invitrogen, L23105). Red blood cells were removed by RBC lysis buffer before staining of mouse peripheral blood samples with the following antibodies: mCD45-FITC (BD Biosciences, 553080), hCD45-PE.Cy7 (BD Biosciences, 560915), CD56-APC-Cy7 (BioLegend, 318308), NKG2A-PE (Miltenyi, 130-114-092), CD16-BV788 (BD Biosciences, 612786), and NKp46-BV421 (BioLegend, 331913). For the analysis of NK cell presence in the lung, mouse lungs were harvested and digested in complete RPMI media supplemented with 0.5 mg/mL of collagenase IV (17104019; Gibco) and 25 U/mL of DNAse I (Sigma, 04716728001) for 45 minutes at 37°C under 225-rpm agitation to obtain a single-cell suspension. The following antibodies were used to stain 1 × 10^6^ cells: HLA-E-PE.Cy7 (BioLegend, 342607), CD56-APC-Cy7 (BioLegend, 318308), NKG2A-PE (Miltenyi, 130-114-092), and CD16-BV788 (BD Biosciences, 612786). Dead cells were excluded by gating on 7-AAD^−^ (BD Biosciences, 559925). All flow cytometry analyses were performed using BD FACSCanto™ and BD LSRFortessa™ (BD Biosciences).

### 2.7 Cytotoxicity assay

Luciferase-GFP expressing tumor cells were seeded at 1 × 10^4^ cells per well in flat-bottom 96-well plates and allowed to adhere overnight. NK cells were added at different effector:target ratios and co-cultured for 24 hours at 37°C with 5% CO_2_. Wells containing only tumor cells were used as a control condition. After 24 hours of incubation, the culture medium was removed, and tumor cells were harvested by using trypsin 0.25%/EDTA 2.21 mM subsequently neutralized by the addition of 3 volumes of DPBS containing 20% of FBS and 0.8% 7AAD before FACS acquisition with BD™ High Throughput Sampler (HTS) BDLSRFortessa™ (BD Biosciences). Specific lysis of tumor cells was calculated as follows: % specific lysis = 100 − (alive tumor cells/alive tumor cells alone) × 100%. Alive tumor cells were defined as 7AAD^−^GFP^+^, and all experimental conditions were performed in technical triplicates. For blocking experiments, NK cells were preincubated at 4°C for 30 minutes with 10 μg/mL of anti-NKG2C mAbs (R&D Systems, Minneapolis, MN, USA; MAB1381) or isotype controls (R&D Systems, MAB0041).

### 2.8 *In vivo* xenograft tumor model

Female human-IL-15 transgenic mice NOD.Cg-Prkdcscid Il2rgtm1Wjl/SzJ (stock no. 030890 NSG-Tg Hu-IL15 from Jackson Laboratory, Bar Harbor, ME, USA) were maintained in specific pathogen-free conditions at the Sainte-Justine Research Centre’s animal core facility. All animal experiments were performed in accordance with protocols approved by the institution’s Institutional Animal Care and Use Committee (#2020-2323) following Good Laboratory Practices for Animal Research. A total of 15 mice were injected intravenously (i.v.) with 1 × 10^5^ MDA-MB-231 cells expressing Luciferase-GFP and HLA-E (HLA-E^+^ MDA-MB-231-GFP^+^Luc^+^). Starting the next day after tumor injection, phosphate-buffered saline (PBS) or 1 × 10^7^ of either WT or *KLRC1* ^KO^ NK cells was administered i.v. weekly for a total of 8 weeks (n = 5 per group). A frozen stock of sorted WT and *KLRC1* ^KO^ NK cells was thawed and expanded for 1 week before the initial injection. The cells were subsequently re-expanded weekly as previously mentioned for 8 weeks until the final injection. Tumor burden was tracked by *in vivo* bioluminescence imaging system (Labeo Technologies, Montréal, QC, Canada). Images were acquired following intraperitoneal administration of 3 mg of XenoLight D-Luciferin (PerkinElmer, Waltham, MA, USA; 122799) per mouse the day before NK cell injection. Image analysis and luciferase quantification were performed using the FIJI distribution of ImageJ using a custom script (14). A volume of 100 μL of blood was collected from the mice’s lateral saphenous vein every 2 weeks for blood FACS analysis.

### 2.9 Statistical analysis

Data are presented as the mean ± standard error of the mean (SEM). Differences between the two experimental groups were assessed by paired and unpaired t-tests, under the assumption of normal distribution of both NK cell surface markers and function in healthy donors. The differences between three or four groups were evaluated using the two-way ANOVA. Mouse survival was analyzed using the log-rank Mantel–Cox test. For regression analyses, the Gompertz function in Prism was used with the N_1_ variable constrained to a value of 100:


$$Y=N_{0}\times e^{\text{ln}\left(\frac{N_{1}}{N_{0}}\right)\times \left(1-e^{-k\times x}\right)}.$$

The beginning of the plateau was characterized as the point where the slope is under 0.3 using the inverse derivative of the Gompertz equation:


$$x=\left(W\left[-ln\left(\frac{N_{1}}{N_{0}}\right)\times e^{ln\left(Y^{\prime }\right)-ln\left(N_{1}\times ln\left(\frac{N_{1}}{N_{0}}\right)\times k\right)}\right]-ln\left(Y^{\prime }\right)+ln\left(N_{1}\times ln\left(\frac{N_{1}}{N_{0}}\right)\times k\right)\right)/k.$$

In order to make the calculations, the main branch of the Lambert W function was approximated as described (15):


W(z)≈2×ln(1+0.8842×2ez+2)−ln(1+0.9294×ln(1+0.5106×2ez+2))−1.2131+1(2×ln(1+0.8842×2ez+2)+4.688).

The 95% CI for the plateau was calculated using the 95% CI of the variables. All statistical analyses were performed using GraphPad PRISM version 9 (GraphPad Software, Inc.), and two-sided *p-*values<0.05 were considered statistically significant.

## 3 Results

### 3.1 Generation of *KLRC1* ^KO^ NK cells

**Figure 1:**
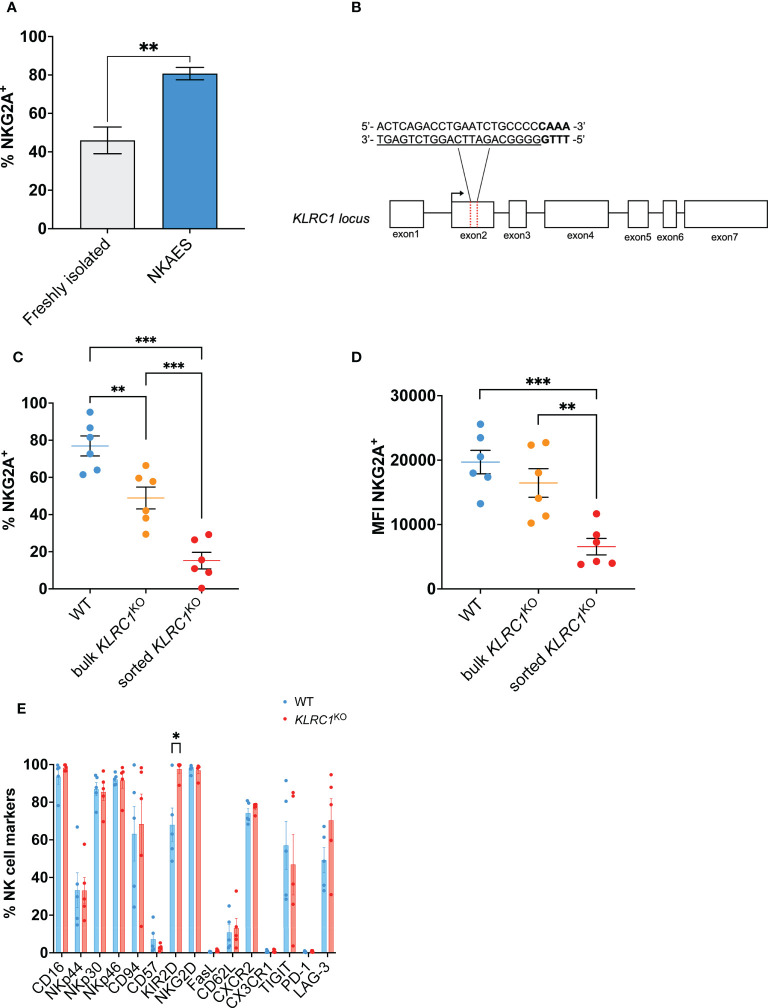
Generation of *KLRC1* ^KO^ NK cells. **(A)** Flow cytometry quantification of the frequency of NKG2A expression among freshly isolated NK cells (n = 3 donors) compared to *ex vivo* expanded NKAES cells (n = 5 donors) (*p* = 0.0020; t-test). **(B)** Schematic representation of CRISPR/MAD7-mediated knockout of *KLRC1* using one guide RNA (gRNA) targeting *KLRC1* gene exon 2. The target sequence is represented as underlined sequence, and PAM is in bold. **(C)** Flow cytometry quantification of the frequency of NKG2A expression among WT, bulk *KLRC1* ^KO^, and sorted *KLRC1* ^KO^ pair-matched NK cells (n = 6 donors) (WT *vs.* bulk *KLRC1* ^KO^ NK cells, *p* = 0.0012, bulk *vs.* sorted *KLRC1* ^KO^ NK cells, *p* = 0.0002, and WT *vs.* sorted *KLRC1* ^KO^ NK cells, *p* = 0.0001; paired t-tests). **(D)** NKG2A expression levels are estimated by MFI in WT, bulk *KLRC1* ^KO^, and sorted *KLRC1* ^KO^ pair-matched NK cells (n = 6 donors) (WT *vs.* bulk *KLRC1* ^KO^ NK cells, *p* = 0.1274, bulk *vs.* sorted *KLRC1* ^KO^ NK cells, *p* = 0.0020, and WT *vs.* sorted *KLRC1* ^KO^ NK cells, *p* = 0.0007; paired t-tests). **(E)** Frequency of expression of a panel of NK cell surface markers estimated by flow cytometry among WT and *KLRC1* ^KO^ pair-matched NK cell populations (n = 5 donors) (WT *vs. KLRC1* ^KO^ NK cells KIR2D expression, *p* = 0.0294, paired t-test). Data in panels A and C–E are presented as mean ( ± SEM). Statistics *p < 0.05, **p < 0.01, ***p < 0.001. MFI, mean fluorescence intensity; NK, natural killer; NKAES, NK Amplification and Expansion System; WT, wild type.

After 1 week of expansion using the irradiated K562-mb-IL21 feeder cell co-culture system, we observed a significant increase in the proportion of NKG2A-expressing NK cells from 46.0% ± 6.3% in human NK cells freshly isolated from the peripheral blood to 80.7% ± 3.3% in expanded NK cells (NKAES) (*p* = 0.0020) (**Figure 1A**). To reduce NKG2A expression and its engagement with HLA-E on tumor cells, we used a CRISPR/Cas system to target *KLRC1*, the gene encoding for NKG2A, in NKAES. We used the Feldan Shuttle, a non-viral method that relies on a peptide-based technology, to deliver a ribonucleoprotein complex targeting exon 2 of the *KLRC1* locus (**Figure 1B**). This approach utilizes cell-penetrating peptides (CPPs), positively charged short peptides that autonomously mediate the internalization of molecules across cell membranes (16). CPPs are combined with endosomolytic peptides, referred to as shuttles, which bind to and transiently destabilize endosomal membranes, enabling efficient *in vitro* protein delivery and overcoming limited cytosolic distribution caused by endosomal sequestration of cargoes (17). Following RNP delivery, we estimated gene editing efficiency at 41.0% ± 4.5% using a T7E1 mismatch detection assay (n = 3).

*KLRC1* deletion led to a significant reduction of the frequency of NKG2A-expressing cells from 76.9% ± 5.4% in WT NK cells to 48.9% ± 5.9% in *KLRC1* ^KO^ NK cells (*p* = 0.0020) (**Figure 1C**). After cell sorting to enrich for NKG2A^−^ NK cells, both the frequency and the mean fluorescence intensity (MFI) of NKG2A were significantly decreased. The frequency of NKG2A^+^ NK cells decreased from 48.9% ± 5.9% in bulk *KLRC1* ^KO^ NK cells to 15.2% ± 4.5% in sorted *KLRC1* ^KO^ NK cells (*p* = 0.0002), and the NKG2A MFI decreased from 16,454.0 ± 2,214.2 in bulk *KLRC1* ^KO^ NK cells to 6,566.5 ± 1,281.0 in sorted *KLRC1* ^KO^ NK cells (*p* = 0.0020) (**Figures 1C, D**). Sorted *KLRC1* ^KO^ NK cells maintained low and stable expression of NKG2A through up to 6 weeks of expansion and exhibited a similar expansion capacity when compared to WT NK cells (**Figures S1A, B**).

We assessed the impact of NKG2A disruption on other NK cell phenotypic markers (**Figure 1E**). There were no significant differences in the percentage of expression of most analyzed markers, except for an increase in the frequency of KIR2D^+^ NK cells from 68.0% ± 8.9% in WT NK cells to 97.6% ± 2.1% in *KLRC1* ^KO^ NK cells (*p* = 0.0294) and of NKG2C^+^ NK cells among *KLRC1* ^KO^ NK cells, as detailed below. The analysis of the expression intensity of those markers showed that the MFIs of CD94 and CD62L were significantly reduced in *KLRC1* ^KO^ compared to WT NK cells (51,780.6 ± 14,650.1 *vs.* 18,525.8 ± 3,732.0, *p* = 0.0452; and 853.6 ± 84.7 *vs.* 671.8 ± 67.7, *p* = 0.0431, respectively) (**Figure S1C**).

In parallel, we used the BaEv pseudotyped lentiviral transduction approach to target *KLRC1* (12). We obtained similar results as with the Feldan Shuttle technique, with a significant decrease in the frequency of NKG2A expressing cells from 86.9% ± 1.0% in WT NK cells to 40.3% ± 9.2% in LV-*KLRC1* ^KO^ NK cells (*p* = 0.0068) (**Figure S1D**). Given the similar efficacy of the Feldan Shuttle and lentiviral system to generate *KLRC1* ^KO^ NK cells, and considering the potential genotoxicity of the latter, we performed our study using Feldan Shuttle gene-edited NK cells.

### 3.2 *KLRC1* ^KO^ cells overcome HLA-E-mediated inhibition of NK cell cytotoxicity

**Figure 2:**
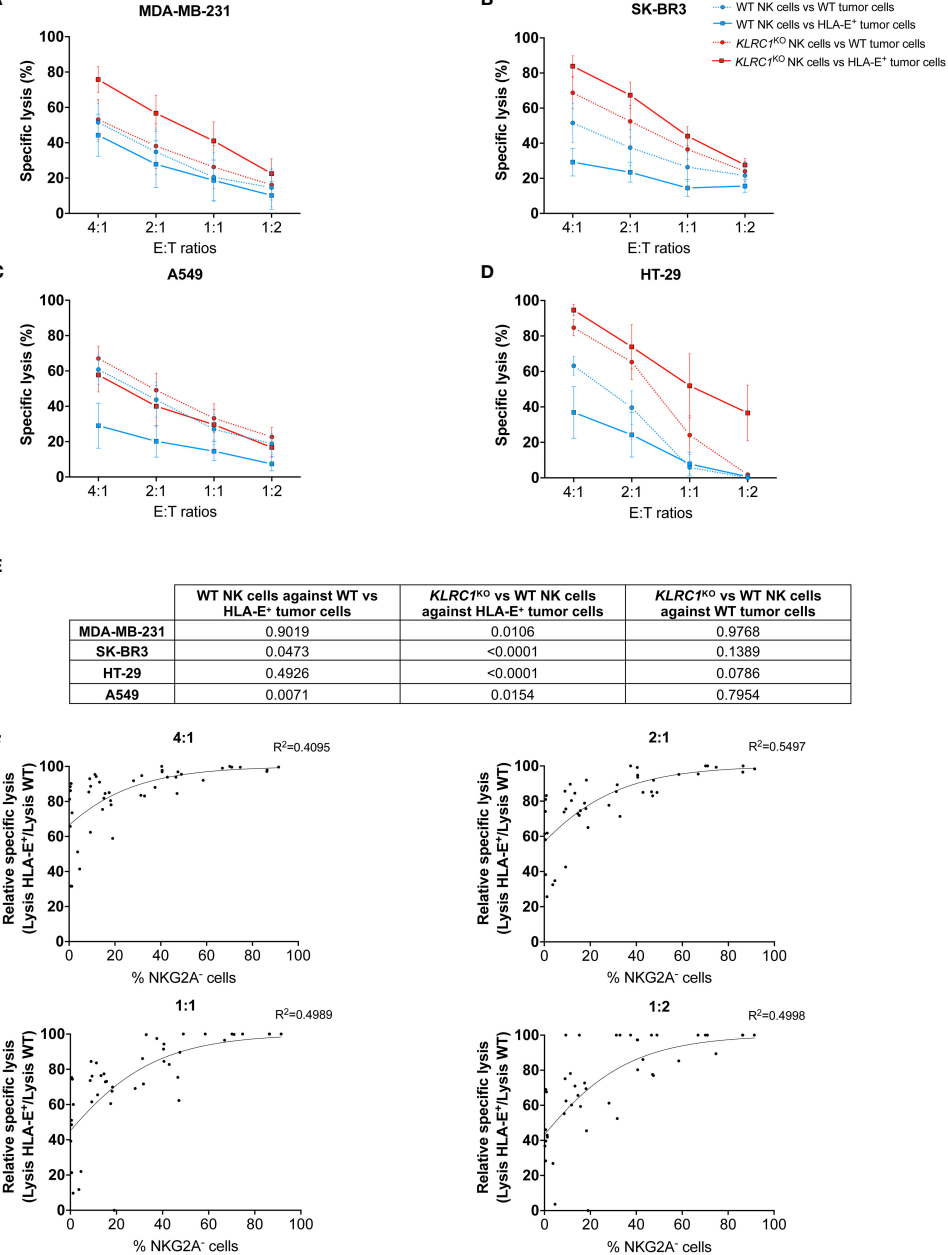
*KLRC1* ^KO^ NK cells overcome HLA-E-mediated inhibition in solid tumors. Cytotoxicity assays of either WT (blue lines) or *KLRC1* ^KO^ NK cells (red lines) against HLA-E^+^ tumor cells (solid lines) or WT tumor cells (dotted lines). **(A)** WT MDA-MB-231 and HLA-E^+^ MDA-MB-231, **(B)** WT SK-BR3 and HLA-E^+^ SK-BR3, **(C)** WT A549 and HLA-E^+^ A549, and **(D)** WT HT-29 and HLA-E^+^ HT-29 were co-cultured with NK cells at the indicated E:T ratios, and cytotoxicity was assessed at 24 hours. **(E)** Table of *p*-values obtained from Tukey’s multiple comparison test after two-way ANOVA for the specific lysis of indicated experimental groups. **(F)** Correlations between the frequency of NKG2A^−^ NK cells and the relative specific lysis at different E:T ratios (non-linear Gompertz fit). Relative specific lysis corresponds to (%lysis *vs.* HLA-E^+^ MDA-MB-231 cells/%lysis *vs.* WT MDA-MB-231 cells) × 100. A value of 100% means no inhibition, while a value of 0% means total inhibition. Data in panels **(A–D)** are shown as mean ( ± SEM) of NK cell-specific lysis isolated from six healthy donors. Data in panel **(F)** are shown as the mean of n = 45 NK cell populations. E:T ratio, effector:target ratio; NK, natural killer; WT, wild type.

To study HLA-E-mediated inhibition of NK cell cytotoxicity, we induced HLA-E expression in four different solid tumor cell lines: the human breast adenocarcinomas MDA-MB-231 and SK-BR-3, the lung carcinoma A549, and the colorectal adenocarcinoma HT-29. While all WT tumors exhibited a similarly low level of HLA-E expression, transducing HLA-E bearing the HLA-Cw1502 signal peptide led to a high frequency of HLA-E^+^ cells ranging from 92.5% to 99.1% depending on the cell line, with a significant increase of the HLA-E MFI between WT and HLA-E^+^ cells (*p* = 0.0095) (**Figures S2A, B**). HLA-E expression on tumor cells reduced NK cell cytotoxicity. Indeed, the cytotoxicity of WT NK cells was significantly decreased against HLA-E^+^ tumors, although it did not reach significance for MDA-MB-231 and HT-29 cells (**Figures 2A–E**). However, knocking out *KLRC1* reversed this phenomenon. Indeed, the cytotoxicity of *KLRC1* ^KO^ NK cells was significantly superior to that of WT NK cells against all HLA-E^+^ tumors across all tested effector-to-target (E:T) ratios (**Figures 2A–E**, **S3A–D**). When facing WT tumors, *KLRC1* ^KO^ NK cells also exhibited enhanced cytotoxicity for SK-BR3 and HT-29, although this difference did not reach significance (*p* = 0.1389 and 0.0786, respectively). Lentiviral-generated *KLRC1* ^KO^ cells also showed increased cytotoxicity against HLA-E^+^ MDA-MB-231, HLA-E^+^ HT-29, and HLA-E^+^ A549 cells compared to WT NK cells, while they were not tested for SK-BR3 cell line (**Figures S4A–C**).

To determine the minimum frequency of NKG2A^−^ NK cells required to overcome HLA-E-mediated inhibition, we assessed the cytotoxic activity of WT and *KLRC1* ^KO^ NK cells with varying levels of NKG2A expression against WT and HLA-E^+^ MDA-MB-231 cells (n = 45). We reported the specific lysis of NK cells against HLA-E^+^ tumors relative to the maximum specific lysis against WT tumors (relative specific lysis). We performed a non-linear regression analysis using the Gompertz curve model to fit the data in relation to the frequency of NKG2A^−^ NK cells for each experiment (**Figure 2F**). We then used a regression formula to determine the plateau values for each effector-to-target ratio (**Table S1**). The calculated plateau values ranged from 43.5% to 60.2% of NKG2A^−^ NK cells required to overcome HLA-E-mediated inhibition of cytotoxicity against MDA-MB-231. These results suggest that a knockout of *KLRC1*, resulting in approximately 50% of NKG2A^−^ NK cells, was sufficient to bypass the HLA-E-mediated inhibition with maximum efficiency.

### 3.3 NKG2C contributes to the improved cytotoxicity of *KLRC1* ^KO^ NK cells against HLA-E^+^ tumors

**Figure 3:**
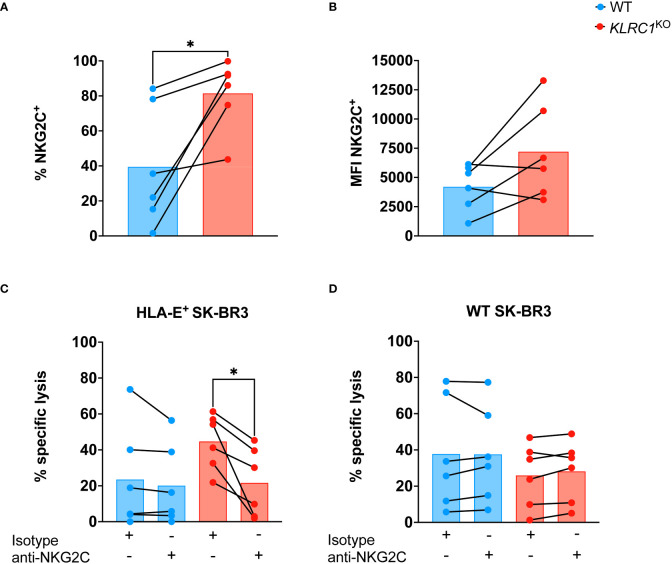
NKG2C contributes to improve *KLRC1* ^KO^ cytotoxicity against HLA-E^+^ tumor. **(A)** Flow cytometry quantification of the frequency of NKG2C expression among WT and *KLRC1* ^KO^ pair-matched NK cells (n = 6 donors) (*p* = 0.0248; paired t-test). **(B)** NKG2C expression levels are estimated by MFI in WT and *KLRC1* ^KO^ pair-matched NK cells (n = 6 donors) (*p* = 0.0749; paired t-test). **(C)** HLA-E^+^ SK-BR3 cells or **(D)** WT SK-BR3 WT cells were cultured with either WT (blue) or *KLRC1* ^KO^ (red) pair-matched NK cells in the presence of either a blocking anti-NKG2C or an isotype control antibody as indicated below the graph, at a 1:1 E:T ratio (n = 6 donors), and cytotoxicity of NK cells was assessed at 24 hours (*p* = 0.0148; paired t-test). Data are presented as repeated measures, individual values are represented by symbols and connected by a line to show paired data across experimental conditions, and bars represent the mean of individual values. Statistics *p < 0.05. MFI, mean fluorescence intensity; E:T ratio, effector:target ratio; WT, wild type; NK, natural killer.

Since NKG2C is the activating counterpart of NKG2A and induces an activating signal upon binding to HLA-E, we assessed the effect of NKG2A disruption on NKG2C expression by NK cells. The frequency of NKG2C^+^ NK cells was found to be significantly higher in *KLRC1* ^KO^ NK cells, increasing from 39.5% ± 13.9% in WT NK cells to 81.4% ± 8.3% in *KLRC1* ^KO^ NK cells (*p* = 0.0248) (**Figure 3A**). We also observed a modest increase in NKG2C MFI, from 4,205.0 ± 1,978.2 in WT NK cells to 7,208.3 ± 4,014.1 in *KLRC1* ^KO^ NK cells, although this difference was not significant (*p* = 0.0749) (**Figure 3B**). To test whether NKG2C interaction with HLA-E could contribute to the improved *KLRC1* ^KO^ NK cell cytotoxicity against HLA-E^+^ tumors, we pre-incubated NK cells with an anti-NKG2C blocking antibody prior to a cytotoxic assay. Although blocking NKG2C did not affect the cytotoxicity of WT NK cells against either WT or HLA-E^+^ SK-BR3 cells, it significantly reduced *KLRC1* ^KO^ NK cell cytotoxicity against HLA-E^+^ SK-BR3 cells (from 44.7% ± 6.3% to 21.6% ± 7.8%, *p* = 0.0148), but not against WT SK-BR3 cells (**Figures 3C, D**). These results strongly suggest that the interaction between HLA-E and NKG2C is at least partially responsible for the enhanced cytotoxicity of *KLRC1* ^KO^ NK cells against HLA-E^+^ tumor cells.

### 3.4 Improved *in vivo* antitumor activity of *KLRC1* ^KO^ NK cells

**Figure 4:**
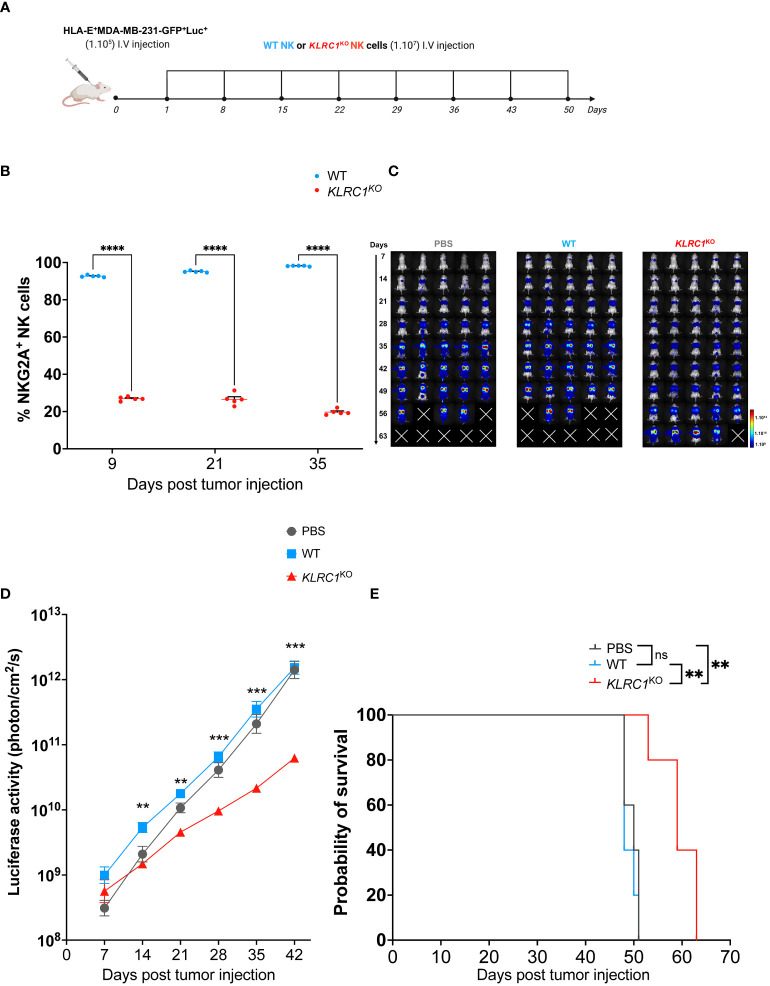
*KLRC1* deletion restores NK cell antitumor activity against HLA-E^+^ tumor *in vivo*. **(A)** Schematic representation of the *in vivo* experiment. Human IL-15 transgenic NSG mice were injected i.v. with 1 × 10^5^ HLA-E^+^ MDA-MB-231-GFP^+^Luc^+^ cells. One day after tumor injection (day 1), PBS, WT, or *KLRC1* ^KO^ NK cells were administered i.v., weekly, for 8 weeks. Tumor burden was monitored by *in vivo* bioluminescence imaging. **(B)** Flow cytometry quantification of the frequency of NKG2A expression among circulating human NK cells found in peripheral blood of WT NK cell-treated mice (n = 5) and *KLRC1* ^KO^ NK cell-treated mice (n = 5) (*p*< 0.0001; two-way ANOVA). **(C)** Representative dorsal images from bioluminescence imaging for all mouse experimental groups over time. **(D)** Quantification of luciferase activity overtime for all mouse experimental groups (n = 5) (WT NK cell-treated mice *vs. KLRC1* ^KO^ NK cell-treated mice, day 14 *p* = 0.0020, day 21 *p* = 0.0012, day 28 *p* = 0.0005, day 35 *p* = 0.0005, and day 42 *p* = 0.0002; two-way ANOVA). **(E)** Kaplan–Meier survival curves of experimental groups (n = 5 mice per group) (untreated *vs. KLRC1* ^KO^ NK cell-treated mice, *p* = 0.0025, and WT NK cell-treated mice *vs. KLRC1* ^KO^ NK cell-treated mice, *p* = 0.0015; two-tailed log-rank Mantel–Cox test). In panels **(B, D)** data are shown as mean ± SEM. Statistics *p < 0.05, **p < 0.01, ***p < 0.001, ****p < 0.0001. NK, natural killer; PBS, phosphate-buffered saline; WT, wild type.

The antitumor activity of *KLRC1* ^KO^ NK cells *in vivo* was evaluated using a xenogeneic metastatic tumor mouse model. Human IL-15 transgenic NSG mice were engrafted with 1 × 10^5^ HLA-E^+^ MDA-MB-231 cells expressing luciferase. Starting the next day after tumor injection, either 1 × 10^7^ sorted WT or *KLRC1* ^KO^ NK cells were administered weekly to mice for a total of 8 weeks (**Figure 4A**). Monitoring the frequency of NKG2A^+^ human NK cells in the peripheral blood of mice showed that *KLRC1* ^KO^ NK cell-treated mice had a significantly lower percentage of NKG2A^+^ human NK cells in the circulation than WT NK cell-treated mice (*p*< 0.0001) (**Figure 4B**). The tumor burden of mice was monitored over time by quantifying luciferase activity using *in vivo* bioluminescence imaging (**Figures 4C, D**). Although WT NK cell-treated mice had similar tumor progression as untreated mice, the administration of *KLRC1* ^KO^ NK cells resulted in delayed tumor growth. *KLRC1* ^KO^ NK cell-treated mice exhibited a significantly reduced tumor burden when compared to WT NK cell-treated mice from day 14, as indicated by a decrease in bioluminescence signal from 5.8 × 10^9^ ± 1.2 × 10^9^ photons·cm^−2^·s^−1^ for the WT NK cell-treated mice to 1.6 × 10^9^ ± 2.5 × 10^8^ photons·cm^−2^·s^−1^ for *KLRC1* ^KO^ NK cell-treated mice (*p* = 0.0020). On day 42, *KLRC1* ^KO^ NK cell-treated mice showed significantly lower tumor burden when compared to both untreated mice and WT NK cell-treated mice (*p* = 0.0010 and *p* = 0.0002, respectively). No difference was observed between untreated and WT NK cell-treated mice. Adoptive transfer of *KLRC1* ^KO^ NK cells significantly improved mice’s survival time, with a median survival of 59 days, in contrast to a median survival of 48 days observed with mice’s WT NK cell administration (*p* = 0.0015) (**Figure 4E**). The presence of human NK cells in mouse lungs that were the primary site of tumor metastasis in our xenogeneic model was assessed by flow cytometry (**Figures S5A, B**). Although NK cells were found in both treated mouse groups, the lungs of WT NK cell-treated mice predominantly showed the presence of NKG2A^+^ NK cells (94.7% ± 1.1%), while *KLRC1* ^KO^ NK cell-treated mice exhibited a lower percentage of NKG2A^+^ NK cells in their lungs (44.7% ± 3.1%) (*p*< 0.0001).

## 4 Discussion

NK cell efficacy in solid malignancies is hindered by the immunosuppressive effects of the TME, and overcoming the negative NK cell regulation is crucial for the development of effective immunotherapy against solid tumors. In this study, we targeted the NKG2A/HLA-E axis by disrupting NKG2A in human NK cells. Our *in vitro* assays demonstrated that *KLRC1* ^KO^ NK cells exhibited an improved cytotoxic function against HLA-E^+^ tumors compared to WT NK cells. This finding translated into improved antitumor activity since *KLRC1* ^KO^ NK cell adoptive transfer to mice bearing an HLA-E^+^ breast tumor resulted in reduced tumor burden and increased survival of mice. In contrast, administration of WT NK cells did not elicit any antitumor response in this mouse model, which highlights the importance of this immune checkpoint in tumor control *in vivo*. In addition to abolishing the NKG2A/HLA-E inhibitory axis, our results indicated a switch toward an HLA-E-mediated enhancement of the NK cell cytotoxicity likely through the interaction with the activating receptor NKG2C, as demonstrated by the anti-NKG2C blocking assay. Indeed, NKG2A and NKG2C compete to form heterodimers with CD94, which then bind to HLA-E (18). Variations in the amino acid residues at the interaction interface between the NKG2 molecules and CD94 result in a sixfold stronger binding affinity of NKG2A for HLA-E compared to NKG2C (19, 20). Hence, in *KLRC1* ^KO^ NK cells, NKG2C no longer competes with NKG2A for the binding of HLA-E, with which it can therefore interact. This activating interaction may explain why knocking out *KLRC1* not only restored cytotoxic function against HLA-E^+^ tumors to the level of WT NK cells against WT tumors but also markedly enhanced it for three out of four tested cell lines. However, the direct impact of the genetic deletion of *KLRC1* in NK cells on the transcriptional regulation of *KLRC2*, encoding for NKG2C, cannot be excluded. We observed also an increase in KIR2D expression following *KLRC1* knockout. Although the underlying mechanisms governing the expression of NKG2A and KIRs are still not fully understood, a negative correlation between these two factors has been suggested (21). While immature NK cells primarily express NKG2A, as they mature, they undergo a transition to co-express KIRs and NKG2A. Ultimately, the most mature NK cells predominantly express KIRs alone. In our study, the *ex vivo* expansion system overstimulates NK cells, leading to increased NKG2A expression. KIR2D upregulation in *KLRC1* ^KO^ NK cells could potentially be explained by a compensatory mechanism that would maintain some inhibitory signaling in the absence of NKG2A expression. However, this increase in KIR2D expression in *KLRC1* ^KO^ NK cells was insufficient to counteract the activation of NK cell cytotoxicity. Interestingly, *KLRC1* ^KO^ NK cells exhibited a modest enhancement in cytotoxicity against WT tumor cell lines as compared to WT NK cells, particularly for SK-BR3 and HT-29. This suggests that NK2GA disruption may unleash NK cell activation independently of HLA-E. NKG2A could mediate inhibitory tonic signaling in continuously activated *ex vivo* expanded NK cells, and the disruption of NKG2A could unleash NK cell activation even in the absence of HLA-E expression by target cells.

Our findings are consistent with those of previous studies showing that disruption of NKG2A, by protein expression blockers or CRISPR/Cas9, results in improved antitumor activity of NK cells against HLA-E^+^ Ewing sarcoma and multiple myeloma, respectively (5, 22). Similar to these previous studies, we did not observe any significant change in the expression of most NK cell phenotypic markers following NKG2A disruption. In contrast with those studies, we did observe an increase in NKG2C expression in *KLRC1* ^KO^ NK cells. One potential reason for this divergence might stem from differences in the method used to expand NK cells. To overcome the challenge of poor NK cell expansion *in vitro*, several *ex vivo* expansion techniques have been developed to generate a clinically significant number of NK cells. These methods rely on IL-2 and IL-15 or IL-21 cytokine-induced proliferation, mostly through cytokine supplementation, or by co-culture system with irradiated feeder cells expressing a membrane-bound form of those cytokines. If the continuous activation supports high proliferation rates, it can also drive phenotypic alterations, adding further heterogeneity in the NK cell populations (23). Herein, we used an IL-21-based expansion system, while previous studies used an IL-15-based one, which may account for the comparable levels of NKG2C expression previously reported between WT and NKG2A-disrupted NK cells. Indeed, while both IL-15 and IL-21 expansion systems have been shown to increase NKG2A expression relative to NKG2C, these cytokines induce distinct downstream activating pathways that could differentially regulate NKG2C expression in NKG2A-engineered NK cells (24). Moreover, high concentrations of IL-15 have been found to induce the downmodulation of NKG2C in NK cells (25).

For now, the use of an *ex vivo* expansion system is indissociable from NK cell clinical application. Among existing ones, the K562-mb-IL21 system used in this study has been shown to provide the highest proliferation rate of NK cells from PBMCs and has been used safely in NK cell clinical trials (1, 26). However, a major drawback of this system is the induction of a high frequency of NKG2A^+^ NK cells upon expansion, as shown here and as previously reported, underscoring the relevance of disrupting NKG2A in such expanded NK cells. Alternatively, the derivation of NK cells from induced pluripotent stem cells (iNK) is a promising strategy to obtain clinically meaningful cell doses. The major advantage of the iNK approach lies in its ability to generate genetically modified cells at a clonal level, which is particularly appropriate for the disruption of negative regulators of NK cells (27). However, given the crucial role of NKG2A in NK cell education, generating effective NK cell therapy from induced pluripotent stem cells (iPSCs) knocked out for *KLRC1* could be challenging. Indeed, during NK cell development, the acquisition of the cytotoxic function depends on the engagement of inhibitory receptors with self-MHC-I molecules, and NKG2A engagement is required by NK cells to become functionally competent (28, 29). Accordingly, previous studies revealed that NKG2A-mediated education is essential to generate mature functional iNK cells (30). Disrupting NKG2A in mature educated NK cells, as reported here, should ensure the preservation of NK cells with optimal cytotoxic function, while this may not be the case in iNK cells.

While interest in CRISPR-based genome editing has grown exponentially in the field of NK cell immunotherapy, the use of this technology complexifies clinical translation due to potential off-target mutations and associated risk for oncogenic transformation (31). To address this limitation, we utilized a Cas12a homologous endonuclease called MAD7, which has a lower off-target effect than Cas9 (32, 33). Furthermore, the delivery of the RNP complex, consisting of MAD7 and the guide RNA targeting *KLRC1*, into NK cells led to transient nuclease activity and further reduced off-target effects (34). To further minimize cellular stress, we utilized the peptide-based Feldan Shuttle for delivery in NK cells, which is less deleterious than nucleofection or viral-based methods (35, 36). However, the strategy we report here would still require careful genomic analysis to ensure safety for clinical application. Alternatively, non-genomic disruption of NKG2A, using shRNA or endoplasmic reticulum-retention domains, as previously reported, could be considered a safer approach than CRISPR (5, 37). Even though those methods have less efficiency in disrupting NKG2A compared to genetic deletion, they may be sufficient in the context of NK cell-based therapy since our results suggest that only 50% of NKG2A^−^ cells among engineered-NK cells are sufficient to completely overcome HLA-E-mediated inhibition.

The findings of the present study must be seen in light of some limitations. Although HLA-E expression on the cancer cell surface is increased in response to IFN-γ in the TME, in our study, we transduced all solid tumor cell lines with HLA-E plus HLA-Cw1502 signal peptide to overcome the low expression of HLA-E on *in vitro* cultured cells (38). Our strategy enabled high and stable levels of HLA-E expressed on the surface of all cancer cell lines, whereas HLA-E level could be cell-line dependent upon exposure to IFN-γ (39). Certain limitations should be considered concerning the use of a single peptide as a simplified representation of reality. Indeed, we used a canonical MHC-I-derived peptide ligand to be presented by transgenic HLA-E, which binds both NKG2A and NKG2C with strong affinity (40). However, the variety of peptide-loaded HLA-E molecules recognized by NKG2C is much more restricted than those recognized by NKG2A. Accordingly, the importance of NKG2C in *KLRC1* ^KO^ NK cell cytotoxicity against HLA-E^+^ tumors reported here could be impaired by alternative HLA-E bound peptides with less affinity for NKG2C. Hence, the transition from NKG2A-mediated cell protection to NKG2C-induced cell lysis might be dependent on the actual HLA-E peptidome of the tumor. Another limitation is the modest, although significant, improvement in survival observed in *KLRC1* ^KO^ NK cell-treated mice. Indeed, tumor control stopped only 1 week after the end of the NK cell injections, which may suggest that *KLRC1* ^KO^ NK cells have a very short life span *in vivo*, despite the presence of human IL-15 in the transgenic mice used. The presence of NK cells with a lower percentage of NKG2A^+^ cells in *KLRC1* ^KO^ NK cell-treated mice compared to WT NK cell-treated mice in lung metastasis is encouraging. However, further investigations will be necessary to study the infiltration and recruitment of *KLRC1* ^KO^ NK cells within the tumor microenvironment. Additional work is needed to characterize *KLRC1* ^KO^ NK cell behavior *in vivo* and optimize the conditions of administration accordingly to improve their antitumor effect.

The present study supports that CRISPR-mediated knockout of *KLRC1* in human NK cells can improve their cytotoxicity against HLA-E^+^ tumors and represents a promising strategy to enhance NK cell-based immunotherapy for solid malignancies. Our finding regarding NKG2C engagement with HLA-E in the absence of NKG2A reinforces the central role of the balance between inhibitory and activating signals in NK cell cytotoxic function regulation. To further improve the antitumor effect of *KLRC1* ^KO^ NK cells, combinations with other strategies could be of clinical significance. For instance, a combination with a CAR molecule directed against a solid tumor antigen will be evaluated in future investigations.

## Data availability statement

The raw data supporting the conclusions of this article will be made available by the authors, without undue reservation.

## Ethics statement

The ethical approval was obtained by the institutional review board (IRB) of the Centre Hospitalier Universitaie (CHU) Sainte-Jusitne (protocol #CER-2019-1956). Healthy volunteers gave their informed written consent prior to the participation in this study.

## Author contributions

AM, DeG, WL, RD, MH, and L-JB performed the experiments and/or analyzed the data. MH, L-JB, DaG, KB, EH, and AM generated the hypotheses and interpreted the data. L-JB, MH, and DaG developed and optimized the delivery method and produced the MAD7. AM, KB, and EH wrote the manuscript. MH and EH conceptualized the study.
