# Supplementary material for: KLRC1 knockout overcomes HLA-E-mediated inhibition and improves NK cell antitumor activity against solid tumors
Source: Front Immunol. 2023 Aug 21;14:1231916. doi: 10.3389/fimmu.2023.1231916 (PMC10478211; doi:10.3389/fimmu.2023.1231916)
Supplement: Supplementary file 1 [file DataSheet_1.docx]

**Supplemental Figures**

**Figure S1. Characterization of *KLRC1*^KO^ NK cells**

**(A)** Flow cytometry quantification of the frequency of NKG2A expression among WT or *KLRC1*^KO^ pair-matched NK cells (n=6 donors) over 6 weeks of expansion after cell sorting. **(B)** Fold expansion of WT or *KLRC1*^KO^ pair-matched NK cells (n=6 donors) over 6 weeks after cell sorting. **(C)** Flow cytometry quantification of the expression levels of NK cell markers as estimated by MFI among WT and *KLRC1*^KO^ pair-matched NK cells (n=5 donors). (WT vs *KLRC1*^KO^ NK cells CD94 MFI, *p*= 0.0452 and CD62L MFI, *p*= 0.0431; paired t-test). **(D)** Flow cytometry quantification of the frequency of NKG2A expression among WT (n=2 donors) and lentiviral-generated *KLRC1*^KO^ NK cells (n=5 donors) (WT vs LV-*KLRC1*^KO^ NK cells, *p*=0.0068; t-tests). Data in A-D are presented as mean (± SEM). Statistics **p*<0.05, ***p*<0.01. MFI, Mean Fluorescence Intensity.

**Figure S2. Generation of HLA-E overexpressing solid tumor cell lines**

Flow cytometry quantification of **(A)** the frequency of HLA-E^+^ cells and **(B)** HLA-E level of expression as estimated by MFI among WT and HLA-E^+^ tumor cell lines (WT vs HLA-E^+^ cell lines, *p*= 0.0095; t-test). Statistics ***p*<0.01. MFI, Mean Fluorescence Intensity.

**
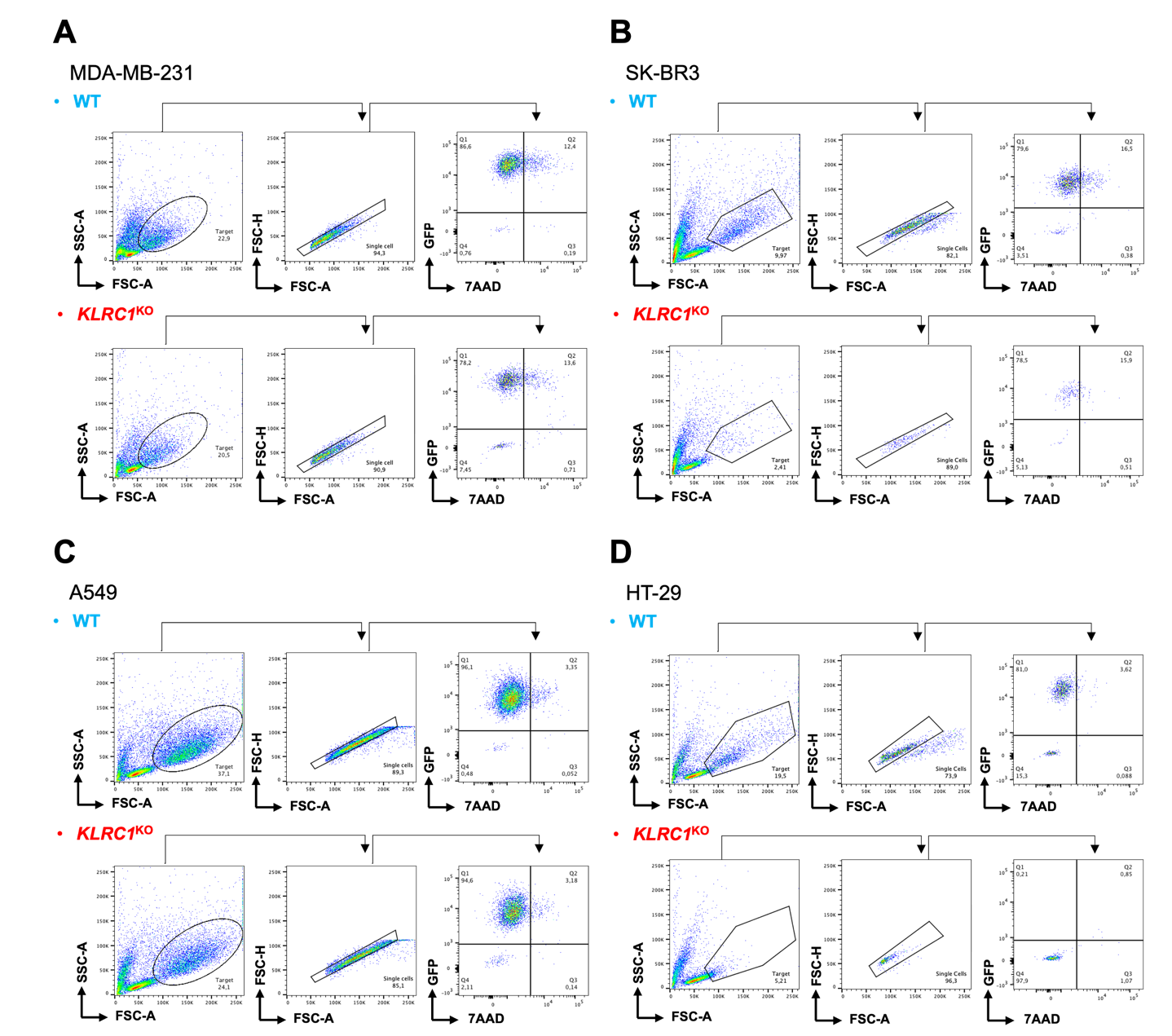
**

**Figure S3. Representative flow cytometry dot plots of NK cell *in vitro* cytotoxic assays**

A representative sample of **(A)** MDA-MB-231, **(B)** SK-BR3, **(C)** A549 and **(D)** HT-29 tumor cells co-cultured with either WT (upper line) or *KLRC1*^KO^ (lower line) NK cells at the 4:1 ratio. Specific lysis was calculated as follows: % specific lysis = 100 - (alive tumor cells/alive tumor cells alone) x 100%. The gating strategy for the detection of alive target cells involved initial gating using a forward scatter (FSC-A) versus side scatter (SSC-A) plot, followed by discrimination of doublets using an FSC-A versus forward scatter height (FSC-H) plot. Alive tumor cells were gated as 7AAD^-^ GFP^+^ events.

**Figure S4. Lentiviral CRISPR/Cas9-modified *KLRC1*^KO^ NK cells overcome HLA-E mediated inhibition in solid tumors**

Cytotoxicity assays of either WT (blue lines) (n=2) or LV-*KLRC1*^KO^ NK cells (red lines) (n=6) against HLA-E^+^ tumor cells (solid lines) or WT tumor cells (doted lines). **(A)** WT MDA-MB-231 and HLA-E^+^ MDA-MB-231, **(B)** WT A549 and HLA-E^+^ A549, **(C)** WT HT-29 and HLA-E^+^ HT-29, were co-cultured with NK cells at the indicated E:T ratios and cytotoxicity was assessed at 24 hours. E:T ratio, effector:target ratio.

**Figure S5. NK cell presence in lung metastasis of HLA-E^+^ breast cancer xenogeneic mouse model**

Representative flow cytometry dot plot graphs showing the gating strategy to assess the presence of NK cells and their expression of NKG2A within the lung tissues of **(A)** WT NK cell-treated mice (n=5) or **(B)** *KLRC1*^KO^ NK cell-treated mice (n=4). On the left panel, the gate selected the alive (7AAD^-^) non tumoral (HLA-E^-^, GFP^-^) cell population. Then, on the middle panel, the CD56^+^CD16^+^ allows to gate on NK cells and assess the frequency of NKG2A^+^ cells (right panel) (94.7 ± 1.1% of NKG2A^+^ NK cells in WT NK cell-treated mice versus 44.7 ± 3.1% in *KLRC1*^KO^ NK cell-treated mice, *p*<0.0001; t-test).

**Table S1. Regression parameters for MDA-MB-231**

| Ratio | 4 | 2 | 1 | 0.5 |
| --- | --- | --- | --- | --- |
| N_0_  (95% CI) | 66.5  (57.5-74.9) | 56.8  (48.4-64.7) | 45.1  (33.9-56.0) | 43.3  (31.7-54.5) |
| K  (95% CI) | 0.0431  (0.0225-0.0784) | 0.0420  (0.0262-0.0655) | 0.0421  (0.0251-0.0685) | 0.0432  (0.0257-0.0696) |
| Plateau (95% CI) | 43.5  (28.3-65.9) | 51.8  (36.9-73.0) | 59.8  (40.2-90.4) | 60.2  (40.5-91.4) |
